# From Margins to Mainstream: Making Psychological Interventions for the Treatment of Obesity and Weight Management Core to NHS Primary Care

**DOI:** 10.7759/cureus.91529

**Published:** 2025-09-03

**Authors:** Hasan Basri, Marwah Al-Waadh, Zeid Mohammed

**Affiliations:** 1 General Practice/Family Medicine, Bishops Close Medical Practice, Durham, GBR; 2 General Practice/Family Medicine, Norton Medical Centre, Stockton-on-Tees, GBR; 3 Psychiatry, Cumbria, Northumberland, Tyne and Wear NHS Foundation Trust (CNTW), Newcastle, GBR

**Keywords:** national health service (nhs), obesity, primary health care, psychology therapy, weight loss and obesity

## Abstract

Obesity continues to place an escalating burden on the NHS, both economically and socially. Despite the introduction of a tiered model of care, psychological support within Tier 2 services remains patchy and is often delivered by non-specialists, thereby limiting its effectiveness. This editorial argues that meaningful progress requires a stronger integration of evidence-based psychological interventions, such as Cognitive Behavioural Therapy, Motivational Interviewing, and Acceptance and Commitment Therapy, into routine primary care. Current provision is highly variable, with many patients facing long waits for higher-tier services and little access to structured mental health input at the community level. Digital tools show promise, but adoption remains inconsistent and inequitable. Primary care is uniquely positioned to address these shortcomings: upskilling GPs and allied staff, embedding psychologists within Primary Care Networks, and scaling digital support could provide earlier, more equitable intervention while reducing the burden on secondary care. If obesity is to be managed effectively and sustainably, Tier 2 services must evolve from their current fragmented state into a model where psychological care is recognised as central, not peripheral, to treatment.

## Editorial

Introduction

Obesity represents a major public health challenge, with NHS data showing that almost two-thirds of adults in England are living with overweight or obesity, and around one in four are classified as obese [1]. Obesity is strongly associated with type 2 diabetes, cardiovascular disease, musculoskeletal conditions, and several cancers, and the resulting morbidity, mortality, and economic impact place a substantial burden on the NHS [1]. In response to the growing cost of obesity on the health service, a tiered approach to obesity management was implemented, which emphasises early detection, individualised intervention, and long-term support [1].

The tiered system comprises four different tiers, ranging from public health interventions in Tier 1 to highly specialised interventions, such as bariatric surgery and other complex surgical procedures, in Tier 4. Tier 2 services target individuals with moderate obesity by delivering a structured lifestyle programme that includes brief psychological interventions, such as Cognitive Behavioural Therapy (CBT) and motivational interviewing (MI) [2]. However, recent studies showed significant variability in the quality and delivery of these psychological interventions across different parts of England, with many services relying predominantly on community nurse-led or life coach-delivered interventions rather than specialist psychological input [3]. The inconsistency in the delivery of services, combined with the extended waiting times for the more specialised Tier 3 and Tier 4 services and the limited access to dedicated mental health professionals in primary care, underscores the need for enhanced training of primary care staff and increased recruitment of psychologists and specialist mental health nurses to provide more robust, community-based interventions. While expanding the healthcare workforce may seem costly, it pales in comparison to the estimated £6 billion annual burden that obesity imposes on the NHS through both direct and indirect costs [4]. Targeted efforts to reduce obesity rates could lead to considerable savings in the long run, as well as improved health-related outcomes across the population [5].

This article reviews the status of obesity management in the NHS, examines the available range of psychological interventions, and proposes strategies to enhance service delivery and improve patient outcomes.

The current guidelines for the management of obesity

The current National Institute for Health and Care Excellence (NICE) guidelines for the management of obesity in primary care focus on early detection, personalised treatment, and support. NICE advocates a stepped-care model where Tier 1 services focus on general health promotion, Tier 2 services (BMI 30-40 or 27-40 with comorbidities) deliver structured weight management, and Tier 3 services offer intensive multidisciplinary support to those who have severe obesity (BMI >40 or >35 with comorbidities). Additionally, Tier 4 services offer direct access to bariatric surgery in people with severe obesity where other interventions failed to achieve meaningful weight loss [1]. The delivery of psychological interventions within Tier 2 services is considerably variable, as several programmes depend on short-term interventions conducted by life coaches and nurses instead of specialist practitioners [3,6].

The psychological interventions used for the management of obesity

Psychological interventions are an essential component of obesity management; they target the cognitive, emotional, and behavioural aspects of obesity. There are several psychological interventions that are used to manage obesity, such as CBT and MI. CBT helps patients identify and modify behaviours and thought patterns that lead to weight gain [1]. MI is a well-established intervention that enhances patients' intrinsic motivation and reduces reluctance toward change [2]. Recently, third-wave interventions, such as Acceptance and Commitment Therapy (ACT) and Mindfulness-Based Cognitive Therapy (MBCT), have gained attention due to their emphasis on acceptance of negative experiences and mindfulness. These techniques can reduce emotional eating and support sustained behavioural change [7]. Additionally, Interpersonal Psychotherapy (IPT) has a potential role in cases where weight gain is linked to interpersonal and emotional difficulties [3].

Weight management services and the challenges

The NHS structured tiered approach to obesity management includes primary care interventions (Tiers 1 and 2) focused on individuals with moderate obesity and specialist services for severe cases (Tiers 3 and 4). Tier 2 services offer structured lifestyle programmes integrating dietary advice, physical activity, and brief psychological interventions. The delivery quality of these services varies widely across regions, along with inconsistent access to multidisciplinary teams and specialist psychological support. Patients with severe obesity (BMI ≥40 or BMI between 35 and 40 with additional comorbidities) receive specialist intensive care through Tier 3 services, but they often face long waiting times because of restricted service capacity and limited resources. Bariatric surgery, as part of Tier 4 services, faces additional challenges such as longer waiting times and inconsistent post-operative support across different regions [1,3,6]. For example, the Royal Devon NHS Trust reported waiting times of up to 66 weeks for Tier 3 services [8]. In Mid and South Essex, a surge in referrals led to a temporary suspension of all new Tier 3 referrals in early 2024 due to increased demand that exceeded capacity [9]. Access to Tier 4 services is even more constrained; approximately 8,000 individuals are currently on waiting lists for bariatric surgery in England, with waiting times of up to two years in some hospitals [10].

The "digital" interventions

Regional disparities in resource allocation and local commissioning practices contribute to an inconsistent provision of psychological support. While some regions have developed robust integrated weight management programmes, others face resource constraints that limit the availability of specialist psychological expertise [3]. Emerging digital and hybrid models, such as online CBT modules and telehealth consultations, are increasingly viewed as promising strategies to expand access and standardise psychological interventions across primary care settings. There is growing evidence that digital psychological interventions can play a valuable role in managing obesity in primary care. For instance, participants in the NHS Digital Weight Management Programme lost an average of 3.9 kg over 12 weeks, with a promising engagement from people in deprived communities and ethnic minority groups [11]. These outcomes are comparable to those seen in face-to-face services and were recognised by NICE as a practical, scalable way to reduce pressure on specialist services [12].

Digital interventions also present certain limitations. Engagement rates can be variable, with some patients discontinuing programmes prematurely [11]. Technological literacy and access to reliable internet remain barriers for older adults, socioeconomically disadvantaged groups, and those with limited digital skills [12]. Furthermore, while digital tools can deliver structured behavioural strategies, they may be insufficient to address more complex psychological needs, such as severe emotional eating or comorbid mental health disorders, where specialist input remains essential [13].

What else can be done

Although psychological interventions for obesity management are recommended within the NHS tiered care framework, the practical availability and intensity of these interventions in primary care remain variable [13]. To improve the effectiveness of obesity management in the primary care setting, it may be beneficial to expand the training of healthcare professionals, such as nurse practitioners and allied health professionals, to deliver psychological interventions [1,6]. High-intensity psychological interventions could be beneficial for patients with lower BMIs who do not meet the criteria for Tier 3 services, as evidence suggests that their implementation is effective in achieving meaningful weight loss [5]. Furthermore, recruiting additional psychologists and mental health nurses at the primary care network (PCN) level to provide community-based Tier 3-like interventions could help bridge the current gap in specialist support within primary care settings.

However, these recommendations must be considered in light of the current national shortage of mental health professionals and the existing heavy workload on primary care teams. GPs already face significant pressures managing multimorbidity and routine care [3], meaning that without additional resources and workforce planning, integrating more psychological support risks overburdening staff. Previous research has also shown that nurse-led interventions can be effective but are limited by workforce availability [13]. Addressing these workforce constraints is therefore critical if psychological interventions are to be embedded sustainably at scale.

Such a multidisciplinary approach in tackling obesity has been shown to not only improve behavioural outcomes but also improve patient adherence, thereby enhancing service efficacy [13]. Integrating such expertise into primary care could reduce the waiting times associated with specialist Tier 3 services and, as a result, lead to significant long-term savings for the NHS. Nonetheless, these potential savings must be weighed against substantial logistical and financial barriers to implementation. Commissioning additional posts for psychologists and mental health nurses, delivering widespread staff training, and scaling digital tools all require upfront investment and coordinated planning at the Integrated Care Board (ICB) level [3]. Without adequate funding and infrastructure, the ambition to expand psychological interventions in primary care may remain limited despite a strong clinical rationale. Early intervention can potentially reduce the rate of progression to severe obesity and therefore reduce the burden of obesity-related complications on the health service.

Conclusion

GPs are well placed to take a vital role in the management of obesity. With proper training and funding at the PCN and ICB level, GP-led services could offer dedicated support and reduce the need for hospital referrals. This approach aligns with the NHS Long Term Plan because it brings the care closer to patients and eases the burden on secondary care. Obesity is too widespread to manage solely in hospitals; it needs to be addressed where most patients already are, which is within the community, with their GP.

In summary, the management of obesity within the NHS continues to evolve; however, current service provision remains patchy. NICE recommends a comprehensive, tiered approach that includes dietary, physical activity, and psychological interventions, but the delivery of robust psychological support in primary care remains inconsistent [1,6]. Limited access to specialists and reliance on brief interventions by non-specialists highlight the urgent need to upskill primary care staff and expand recruitment of psychologists and mental health nurses [3].

At the same time, it is important to acknowledge the practical barriers to implementing these solutions. Digital tools show promise but suffer from variable patient engagement and cannot fully replace specialist care [11]. The national shortage of mental health professionals, combined with the already heavy workload in primary care, poses significant workforce challenges [13]. Furthermore, commissioning new posts, scaling digital programmes, and delivering widespread training all require upfront investment and careful system-level planning [3].

By recognising both the opportunities and the barriers, policymakers can pursue a realistic strategy: one that builds capacity gradually, ensures equitable access, and embeds psychological care into primary care services in a sustainable way. If these challenges are addressed, psychological interventions can move from the margins to the mainstream of obesity management, improving outcomes for patients and delivering long-term value to the NHS.
